# Purified Brominated Indole Derivatives from *Dicathais orbita* Induce Apoptosis and Cell Cycle Arrest in Colorectal Cancer Cell Lines

**DOI:** 10.3390/md11103802

**Published:** 2013-10-11

**Authors:** Babak Esmaeelian, Kirsten Benkendorff, Martin R. Johnston, Catherine A. Abbott

**Affiliations:** 1 School of Biological Sciences, Flinders University, GPO Box 2100, Adelaide, SA 5001, Australia; E-Mail: esma0007@flinders.edu.au; 2 Marine Ecology Research Centre, School of Environment, Science and Engineering, Southern Cross University, GPO Box 157, Lismore, NSW 2480, Australia; E-Mail: kirsten.benkendorff@scu.edu.au; 3 Flinders Centre for Nanoscale Science and Technology, School of Chemical and Physical Sciences, Flinders University, GPO Box 2100, Adelaide, SA 5001, Australia; E-Mail: martin.johnston@flinders.edu.au; 4 Flinders Centre for Innovation in Cancer, Flinders University, Adelaide, SA 5001, Australia

**Keywords:** colorectal cancer, apoptosis, marine mollusc, brominated indoles

## Abstract

*Dicathais orbita* is a large Australian marine gastropod known to produce bioactive compounds with anticancer properties. In this research, we used bioassay guided fractionation from the egg mass extract of *D. orbita* using flash column chromatography and identified fractions containing tyrindoleninone and 6-bromoisatin as the most active against colon cancer cells HT29 and Caco-2. Liquid chromatography coupled with mass spectrometry (LCMS) and ^1^H NMR were used to characterize the purity and chemical composition of the isolated compounds. An MTT assay was used to determine effects on cell viability. Necrosis and apoptosis induction using caspase/LDH assay and flow cytometry (PI/Annexin-V) and cell cycle analysis were also investigated. Our results show that semi-purified 6-bromoisatin had the highest anti-cancer activity by inhibiting cell viability (IC_50_ = ~100 µM) and increasing caspase 3/7 activity in both of the cell lines at low concentration. The fraction containing 6-bromoisatin induced 77.6% apoptosis and arrested 25.7% of the cells in G2/M phase of cell cycle in HT29 cells. Tyrindoleninone was less potent but significantly decreased the viability of HT29 cells at IC_50_ = 390 µM and induced apoptosis at 195 µM by increasing caspase 3/7 activity in these cells. This research will facilitate the development of these molluscan natural products as novel complementary medicines for colorectal cancer.

## 1. Introduction

Colorectal cancer (CRC) is the third most diagnosed cancer worldwide [1] with an incidence of 1.2 million new cases (9.7% of all cancers excluding non-melanoma skin cancers) and 608,000 deaths in 2008 [2]. Many therapeutic strategies are used to fight CRC. However, chemotherapy with drugs such as *5-*fluorouracil and radiotherapy can expose patients to troublesome side effects [3]. Surgical treatment of CRC is associated with a high mortality and the risk of local repetition [4].

Natural products have served as the most productive source of leads for drug development for centuries [5]. In recent decades, many of the new antibiotics and new antitumor drugs approved by the US Food and Drug Administration (FDA), or comparable entities in other countries, are natural products or derived from natural products [6,7,8]. Protective effects against a wide range of cancers, including colon cancer, have been shown by several foods such as nuts, spices, grains, fruits, cereals, vegetables, herbs, as well as medicinal plants and their various bioactive constituents including flavonoids, alkaloids, phenolics, carotenoids, and organosulfur compounds [9]. Natural products are usually considered to exhibit low toxicity, and are cost effective and socially acceptable alternatives to pharmaceutical chemopreventatives [10]. The marine environment is one of the major sources for novel natural products. The immeasurable chemical and biological diversity of the ocean offers a great source for new as yet undiscovered potential bioactive compounds [11,12,13]. Many marine secondary metabolites have shown bioactivity for application as anticancer agents [14,15,16].

The Muricidae (Neogastropoda) are a family of predatory marine gastropods that are historically known for the production of Tyrian purple (6,6′-dibromoindigo), an ancient dye, *de novo* biosynthesized from a choline ester precursor salt of tyrindoxyl sulphate after a series of oxidative, enzymatic and photochemical reactions in the hypobranchial gland and egg masses [17,18,19,20,21]. Tyrindoleninone is the main indole precursor found in the extracts, along with 6-bromoisatin, a natural oxidative by-product of Tyrian purple synthesis [18,22]. 6,6-dibromoindirubin is a structural isomer of Tyrian purple that can form from the combination of tyrindoleninone and 6-bromoisatin [19,23] and is a minor pigment found in hypobranchial and male reproductive gland extracts of some muricids [19,24]. Benkendorff [25] highlights the fact that all of these brominated indole derivatives in Muricidae molluscs conform to Lipinskis’ rule of five for druglikeness and orally active drugs in humans.

Anticancer properties of egg mass extracts and the isolated brominated indoles from the Australian Muricidae *Dicathais orbita*, have been shown by several studies [25]. The extracts have been tested against a panel of cancer cell lines *in vitro* [26]. Tyrindoleninone and 6-bromoisatin purified from *D*. *orbita* extracts were shown to specifically decrease cell viability of female reproductive cancer cells, rather than freshly isolated human granulosa cells [27]. Furthermore, in a study by Vine *et al.* [28], some substituted isatin derivatives including 6-bromoisatin have been synthesized and show *in vitro* anticancer properties on a range of human cancer cells, including leukemia, lymphoma and colorectal (HCT-116) cell lines. Bioassay guided fractionation of secretions from hypobranchial gland of a Mediterranean Muricidae *Hexaplex (Murex*) *trunculus* showed that 6,6-dibromoindirubin is an inhibitor of protein kinases and efficiently inhibits cell proliferation by selectively targeting glycogen synthase kinase-3 (GSK-3) [29,30]. In an *in vivo* study using a rodent model for colon cancer prevention by administrating the DNA damaging agent azoxymethane, pro-apoptotic activity of a crude extract from *D. orbita* containing these brominated indoles, was demonstrated in the distal colon [22]. However, the compound or compounds responsible for the anticancer *in vivo* and *in vitro* activity have not yet been characterized.

Muricidae molluscs are subject to a small scale world-wide fisheries industry and are of growing interest in aquaculture [31,32]. Given that these edible molluscs have anticancer properties, there is growing interest in their potential use as a medicinal food for prevention of colon cancer [25,33]. The aim of this study was to perform bioassay guided fractionation on *D. orbita* extracts and to characterize these fractions *in vitro* using cell viability, apoptosis and cell cycle analysis in two human colon adenocarcinoma cell lines, Caco2 and HT29.

## 2. Results and Discussion

### 2.1. Chemical Analysis and Bioassay Guided Fractionation

**Figure 1:**
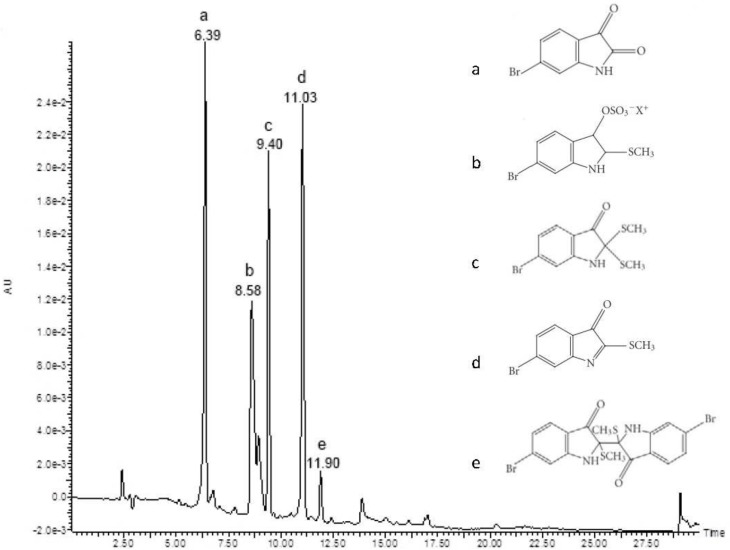
Liquid chromatography-mass spectrometry (LC-MS) analysis of extract from *D. orbita* egg capsules. The chromatogram obtained from diode array detection at 300 and 600 nm shows five peaks corresponding to brominated indoles where a: 6-bromoisatin (*m/z* 224, 226); b: tyrindoxylsulphate (*m*/*z* 336, 338); c: tyrindolinone (*m*/*z* 302, 304); d: tyrindoleninone (*m*/*z* 255, 257) and e: tyriverdin (*m*/*z* 511, 513, 515).

LC-MS analysis of *D. orbita* egg capsule mass crude extract showed five peaks corresponding to brominated indoles (Figure 1). The dominant peak in this extract at *t*_R_ 6.39 min and major ions in ESI-MS at *m*/*z* 224, 226 was attributed to the molecular mass of 6-bromoisatin. Another dominant peak at *t*_R_ 11.03 min corresponded to the molecular weight of tyrindoleninone with major ions at *m*/*z* 255, 257. Mass spectrum of the peak at *t*_R_ 9.40 min with major ions in ESI-MS at *m*/*z* 302, 304 was indicative of tyrindolinone. The peak at *t*_R_ 8.58 min corresponds to tyrindoxyl sulphate, with major ions in ESI-MS at *m*/*z* 336, 338 and a smaller peak at *t*_R_ 11.90 min occurred with ions in ESI-MS at *m*/*z* 511, 513, 515 corresponding to the molecular mass of tyriverdin with major fragment ions at *m*/*z* 417, 419, 421 formed by the elimination of dimethyl disulphide.

**Figure 2:**
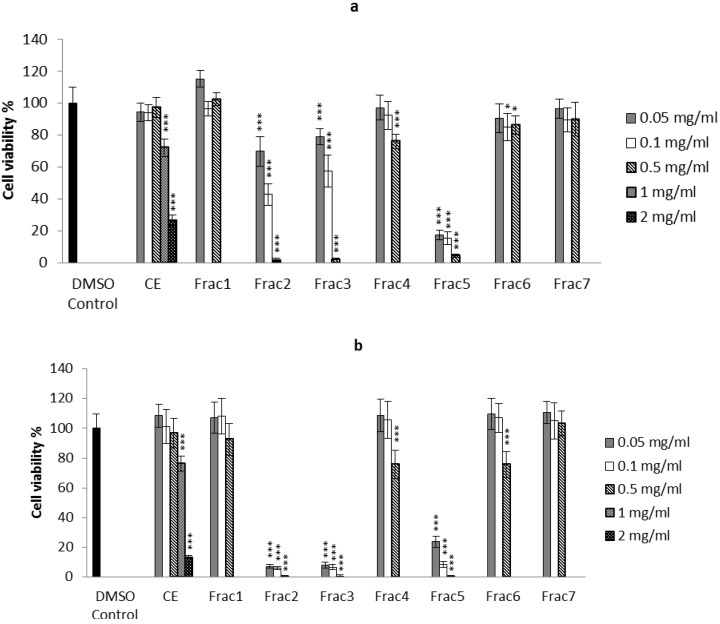
MTT viability results of *D. orbita* egg mass crude extract (CE) and all fractions collected from flash column chromatography (Frac 1–7) on HT29 cells (**a**) and Caco2 cells (**b**). Fraction 1 is the most lipophilic collected with 100% hexane and fraction 7 is the most polar collected with 100% methanol. Significant difference between each group and the 1% DMSO control are shown as *p* ≤ 0.05 (*), *p* ≤ 0.01 (**) and *p* ≤ 0.001 (***).

Bioassay guided fractionation using the 3-(4,5-dimethylthiazol-2-yl)-2,5-diphenyltetrazolium bromide (MTT) cell viability assay revealed a statistically significant mean reduction of 27.6% and 72.4% cell viability in HT29 cells respectively at high concentrations of 1 and 2 mg/mL of crude extract compared with the solvent control (Figure 2a). Caco2 cells showed 86.4% (*p* < 0.001) mean reduction in cell viability when exposed to the highest concentration of crude extract 2 mg/mL (Figure 2b). Significant reductions in cell viability also occurred in some fractions. For example, HT29 cells treated with 0.1 and 0.05 mg/mL of fraction 2, showed 57.3% and 30.2% reduction in formazan production (Figure 2a), while this reduction was more than 90% for Caco2 cells treated with the same concentrations of fraction 2 (Figure 2b). At the highest concentration of 0.5 mg/mL, cell viability was less than 2% in both cell types. Similar activity was observed for fraction 3 (Figure 2). The highest concentration of fraction 4 (0.5 mg/mL) caused 23.9% and 24.3% reduction in cell viability of HT29 and Caco2 cells respectively. Fraction 5 at the concentrations of 0.05 and 0.1 mg/mL showed 76.3% and 91.4% reduction of cell viability for Caco2 cells respectively and the greatest reduction in cell viability for HT29 cells. Fraction 5 reduced the viability of both cell lines by over 95% at the highest concentration of 0.5 mg/mL. A mean reduction of 24.4% in the cell viability of Caco2 cells was also observed in fraction 6 with the higher concentration of 0.5 mg/mL. Significant dose effects were observed in both cancer cell lines, with lower viability rates recorded at the higher treatment concentrations. Bioassay guided fractionation using the MTT assay showed that fractions containing both tyrindoleninone and 6-bromoisatin inhibit the viability of HT29 and Caco2 cells, though tyrindoleninone was more potent towards Caco2 cells than HT29. The effect on viability of fraction 3 (mixture of tyrindoleninone and tyrinolinone) was similar to fraction 2 (tyrindoleninone), indicating the additional methyl thiol group on tyrindolinone does not increase the overall activity.

**Figure 3:**
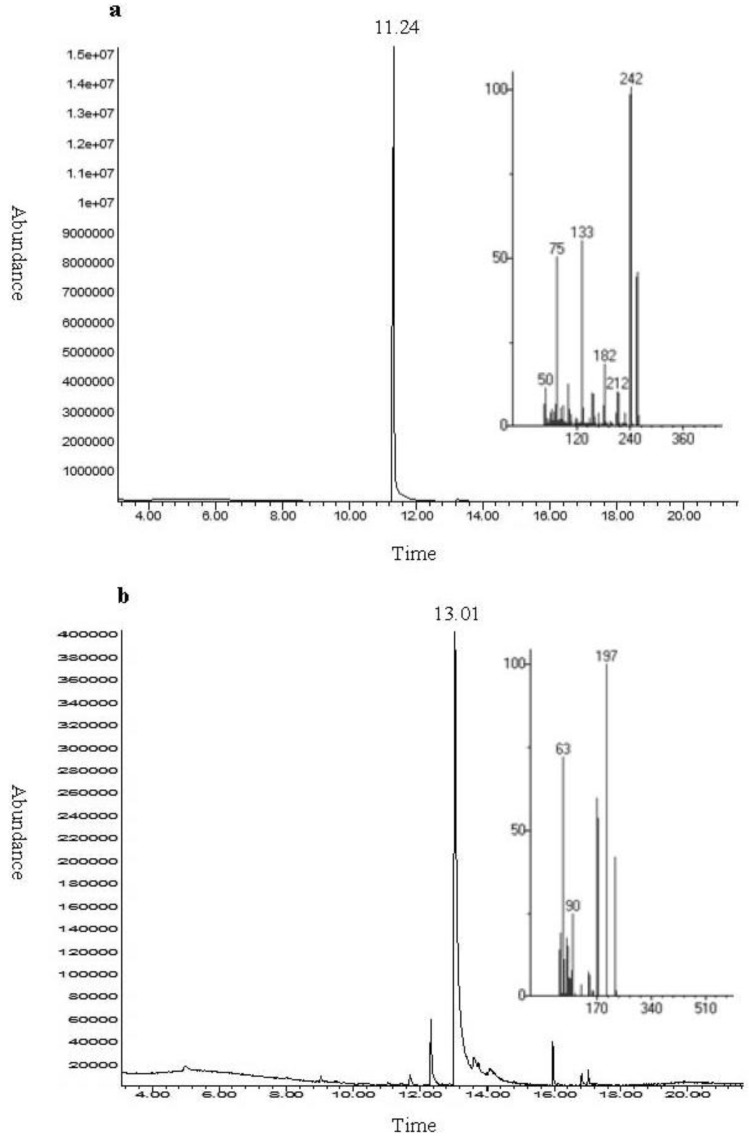
Gas chromatography–mass spectrometry (GC-MS) chromatogram of fractions from the egg masses extract of the Australian muricid, *D. orbita*. Fraction 2 (**a**) at *t*_R_ 11.24 min corresponds to tyrindoleninone and fraction 5 (**b**) with dominant peak at *t*_R_ 13.01 min matches the molecular mass of 6-bromoisatin. The mass spectra (ESI-MS) for the major peaks are inset.

All fractions from flash chromatography of the crude egg capsule extract that were found to effect cell viability using the MTT assay, were then analyzed by LC-MS. In addition to matching the molecular mass of the isolated compounds with tyrindoleninone and 6-bromoisatin, the identity of these compounds was also confirmed by data gained from ^1^H NMR. One purified compound was identified in fraction 2 at *t*_R_ 11.03 min, which was attributed to the molecular mass of tyrindoleninone (*m*/*z* 255, 257). The purity and identity of tyrindoleninone in fraction 2 was confirmed by GC/MS with one peak at *t*_R_ 11.24 min and exact MS match to tyrindoleninone in the mass spectrum library (Figure 3a). ^1^H NMR also confirmed the identity of tyrindoleninone: ^1^H NMR (400 MHz, CD_3_CN) δ 7.46 (^1^H, dd, *J* = 0.5, 1.4 Hz), 7.42 (^1^H, dd, *J* = 0.5, 7.6 Hz), 7.39 (^1^H, dd, *J* = 7.6, 1.4 Hz), 2.63 (3H, s). Our data for tyrindoleninone was consistent with the ^1^H NMR results for this compound previously reported by Benkendorff *et al*. [18] and Baker and Duke [34]. LC-MS of fraction 3 revealed two major peaks at *t*_R_ 9.40 and 11.03 min corresponding to the molecular mass of tyrindolinone (*m*/*z* 302, 304) and tyrindoleninone (*m*/*z* 255, 257) respectively. LC/MS of fraction 5 identified one major compound at *t*_R_ 6.42 min which was indicative of 6-bromoisatin (*m*/*z* 224, 226). GC/MS revealed several other minor compounds (at least six peaks) in this fraction but confirmed 6-bromoisatin as the major component (90%) with a dominant peak at *t*_R_ 13.01 min (Figure 3b). The other minor compounds in fraction 6 were matched with two short chain aldehydes at *t*_R_ 11.71 min and *t*_R_ 12.35 min, two sterols at *t*_R_ 16.82 min (molecular mass of 366% and 93.7% match with cholesta-4,6-dien-3-ol (3β); C_27_H_44_O) and at *t*_R_ 17.02 min (molecular mass of 364), an unidentified ester at *t*_R_ 15.96 min (molecular mass of 302) and finally a new brominated indole with a tiny amount was found at *t*_R_ 13.61 min (molecular mass of 267/269). ^1^HNMR confirmed the identity of the major compound in fraction 5 as 6-bromoisatin: ^1^H NMR (400 MHz, CD_3_CN) δ 8.96 (^1^H, s), 7.44 (^1^H, d, *J* = 8.08 Hz), 7.30 (^1^H, dd, *J* = 1.64, 8 Hz), 7.19 (^1^H, d, *J* = 1.6 Hz) despite also detecting small peaks associated with minor contaminants. Chemical analysis of the most bioactive fractions showed that a good separation for tyrindoleninone producing pure material and a semi-purification for 6-bromoisatin (90% purity) based on the GC/MS analysis.

### 2.2. Biological Activity of the *D. orbita* Compounds

#### 2.2.1. Apoptosis, Necrosis and Cell Viability

Death by necrosis, which may result in damage to the plasma membrane and releasing of the cytoplasmic contents, including lysosomal enzymes into the extracellular fluid, is often considered as a toxic process in comparision to apoptosis [35,36]. The most bioactive fractions from the MTT assay—fraction 2 (tyrindoleninone) and fraction 5 (semi-purified 6-bromoisatin) were examined for their ability to induce either apoptosis or necrosis.

**Figure 4:**
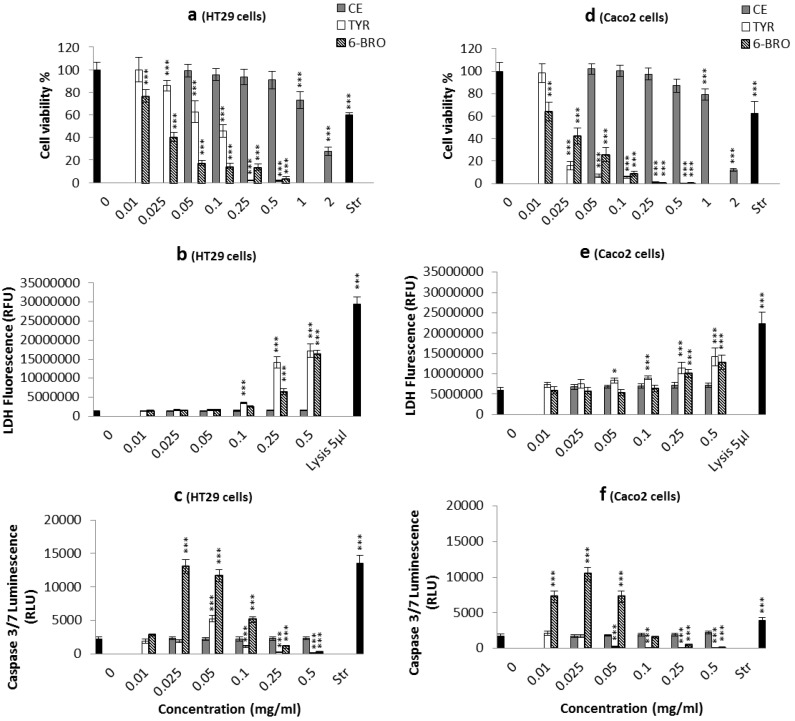
Effects of *D. orbita* egg mass crude extract (CE), purified tyrindoleninone (TYR) and semi-purified 6-bromoisatin (6-BRO) in mg/mL on HT29 (left panels) and Caco2 (right panels) cells. Cell viability (**a**,**d**), lactate dehydrogenase (LDH) release (**b**,**e**) and caspase-3/7 activity (**c**,**f**). LDH release was measured by fluorescence at 535EX/590EM and caspase-3/7 activity was measured at full light on a luminescence plate reader. Staurosporin (Str) (5 µM; Sigma) was used as a positive control for the MTT and caspase-3/7 assay; lysis buffer (5 µL/well; Promega) served as the positive control for the LDH assay. A final concentration of 1% DMSO was used in all control and treated cells. The results are the mean for three independent repeat assays each performed in triplicate (*n* = 3). Significant difference between each group and the DMSO control are shown as *p* ≤ 0.05 (*); *p* ≤ 0.01 (**) and *p* ≤ 0.001 (***).

Tyrindoleninone, was found to be more cytotoxic towards Caco2 cells (IC_50_ = 98 μM), than for the HT29 cells (IC_50_ = 390 μM; Figure 4a,d). In a study by Benkendorff *et al*. [26], greater reduction in cell viability (over 60%) was observed in Caco2 and U937 lymphoma cells treated using a semi-purified egg extract with increased concentration of tyrindoleninone, compared to crude extract, whereas less activity was observed against HT29 cells. This confirms our result that Caco2 cells are more susceptible to tyrindoleninone than HT29 cells. Edwards *et al*. [27] showed that tyrindoleninone inhibited KGN cell viability (a tumour-derived granulosa cell line), JAr and OVCAR-3 cells with the IC_50_ 39 μM, 39 μM and 156 μM respectively. In addition, Vine *et al.* [37] demonstrated that tyrindoleninone had less cytotoxic effects on untransformed human mononuclear cells (IC_50_ = 195 μM) than U937 cancer cells (IC_50_ = 4 μM) after 1 h exposure. The current study confirms the different cell line specificity of tyrindoleninone, with a four-fold difference observed here between the two adherent colon cancer cells lines. This difference in drug resistance may be due to the variations in metabolic and signaling pathways and also the difference in expression and activity of some drug-metabolizing enzymes in different cancer cells [38].

The other bioactive compound, 6-bromoisatin however, inhibited the viability of both Caco2 and HT29 cells (IC_50_ = 100 μM; Figure 4a,d). Edwards *et al.* [27] demonstrated that semi-purified 6-bromoisatin significantly reduced cell numbers of three reproductive cancer cell lines KGN, JAr and OVCAR-3, although converse to this study, it was not as potent as tyrindoleninone. The JAr cells were the most susceptible, with cell numbers halved at approximately 223 μM 6-bromoisatin. Vine *et al.* [28], on the other hand, showed that a range of isatin derivatives including 7-bromoisatin (IC_50_ = 83 μM) and 6-bromoisatin (IC_50_ = 75 μM) reduced the cell viability of lymphoma cell line U937, which was similar to the efficacy of 6-bromoisatin against Caco2 and HT29 cells in our study (IC_50_ = 100 μM). Vine *et al.* [28] also reported different specificity of isatin derivatives against different cancer cell lines. Human leukemic Jurkat cell lines were the most sensitive to isatin treatment (IC_50_ = 5–20.9 µM), the next most sensitive cells were the colon cancer cell line HCT-116 (IC_50_ = 15.9–37.3 μM) and the least sensitive cells were the prostrate PC3 cell line (IC_50_ = 25.9–101 μM). In a review by Vine *et al*. [39], small electron withdrawing groups, mono, di and tri-halogenation at positions 5, 6 and/or 7 on the isatin molecule were found to enhance cytotoxicity activity. 6-Bromoisatin is an example of this kind of halogenated isatin.

**Figure 5:**
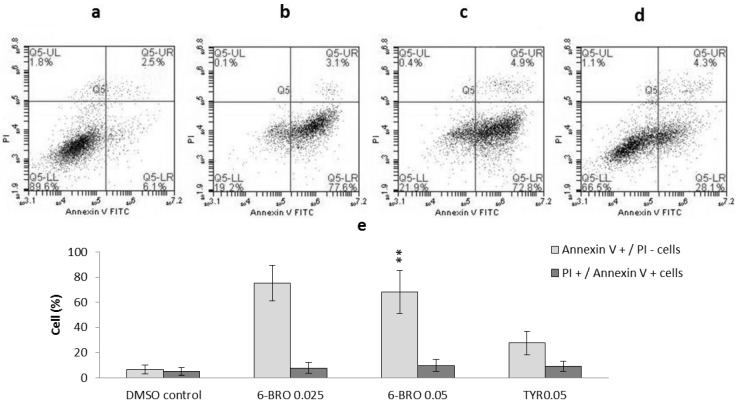
Flow cytometric analysis of HT29 cells (1.5 × 10^5^) treated with (**a**) DMSO only (final concentration 1%); (**b**) 0.025 mg/mL semi-purified 6-bromoisatin; (**c**) 0.05 mg/mL semi-purified 6-bromoisatin and (**d**) 0.05 mg/mL tyrindoleninone purified from *D. orbita* egg masses. Cells were treated for 12 h and stained with Annexin-V-FITC and PI then analyzed by a FACscan flow cytometer and FlowJo analysis software. *X*-axis shows Annexin-V positive cells and *Y*-axis shows propidium iodide (PI) positive cells. (**e**) Histograms of the mean ± SE of three separate experiments for PI and annexin positive cells Significant difference between each group and the DMSO control are shown as *p* ≤ 0.05 (*) and *p* ≤ 0.01 (**).

**Figure 6:**
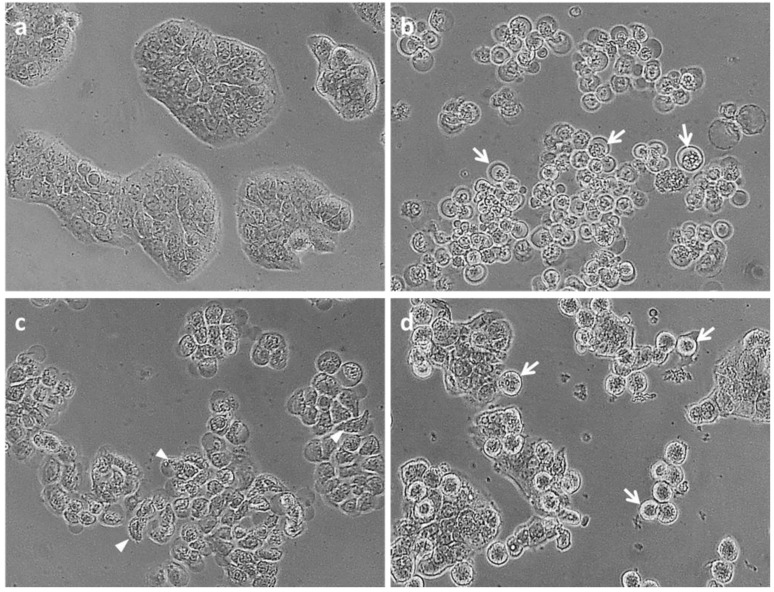
HT29 cells at 400× magnification under an Olympus inverted microscope. DMSO control (**a**); cells treated with 0.05 mg/mL semi-purified 6-bromoisatin (**b**); cells treated with 0.5 mg/mL semi-purified 6-bromoisatin (**c**) and cells treated with 0.05 mg/mL tyrindoleninone (**d**) for 12 h (final concentration of 1% DMSO). Apoptotic cells with chromatin condensation characteristic are shown by arrows and necrotic cells with deformed cell shapes are shown by arrowheads.

Caspase-3 and -7 activity significantly increased only in HT29 cells treated with 195 µM (0.05 mg/mL) tyrindoleninone in 1% DMSO compared to the 1% DMSO control (Figure 4c). An increase in the proportion of Annexin-V positive, PI negative cells (27.6% ± 9.25%) was also observed by flow cytometry in HT29 cells treated with 195 µM (0.05 mg/mL) tyrindoleninone; however, it was not significant (Figure 5). Despite a dose-dependent decrease in viability from Caco2 cells treated with tyrindoleninone, no significant increase in caspase-3 and -7 activity was observed (Figure 4d). Tyrindoleninone at high concentrations appears to induce necrosis rather than apoptosis (increase in LDH observed, Figure 4e) towards Caco2 cells, whereas some apoptosis by caspase 3/7 up-regulation was observed in HT29 treated with tyrindoleninone. Apoptotic cells are characterized by particular morphological features [40,41], such as dense chromatin surrounded by a halo, which were observed in the treated HT29 cells in this study (Figure 6d). Purification of tyrindoleninone from the crude extract consistently increased the cytotoxic potency towards Caco2 cells, but resulted in induction of necrosis rather than apoptosis in these cells, whereas HT29 cells, which were more resilient to the anti-proliferation effects of tyrindoleninone, underwent apoptosis at the concentration of 195 μM. This difference in cell line specificity might be due to the phenotype of the cells, as bioactive compounds may target alternative pathways in different cells [26,42]. Edwards *et al*. [27] revealed that purified tyrindoleninone induced 66% apoptosis with 20 μM in KGN compared to 31% apoptosis (391 μM) in freshly isolated human granulosa cells (HGC) using TUNEL assay after 4 h. This study showed that reproductive cancer cell lines were ten times more susceptible than HCG to tyrindoleninone and indicated specificity of this compound toward reproductive cancer cells.

The fraction containing 6-bromoisatin considerably activated caspase-3 and -7 enzymes and induced cell death by apoptosis in both cell lines at approximately 100 µM (0.025 mg/mL) and 200 µM (0.05 mg/mL), much lower concentrations than those required to cause lactate dehydrogenase (LDH) release and necrosis (~1000 to ~2000 µM; Figure 4b,e). For example, the HT29 cells treated with 6-bromoisatin at ~100 µM and 200 µM showed significant increases in caspase-3 and -7 activity, with luminescence values greater than five times the negative (DMSO) control. The light microscopic images from the HT29 cells treated with ~200 µM 6-bromoisatin showed morphological alterations, such as chromatin condensation characteristic of the apoptotic process (Figure 6b). Flow cytometry results (Figure 5) also confirmed that HT29 cells treated with ~100 µM (0.025 mg/mL semi-purified) 6-bromoisatin underwent a significant induction of apoptosis (75.3% ± 14.03% Annexin-V positive, PI negative cells) compared with the DMSO control (6.6% ± 3.43% Annexin-V positive, PI negative). Similarly, ~200 µM 6-bromoisatin, induced apoptosis up to 68.1% ± 17.1%, but also with a 9.7% increase in the number of PI positive necrotic cells, as compared to DMSO control. In contrast, the highest concentrations of 6-bromoisatin (~1000 µM and 2000 µM) caused a high release of LDH indicating necrosis in HT29 cells (Figure 4b) without any sign of apoptosis. HT29 cells incubated with approximately 400 µM of 6-bromoisatin underwent a significant induction of apoptosis, while the increase in LDH release did not reach significance at this concentration (Figure 4b). Caco2 cells treated with the three lowest concentrations of semi-purified 6-bromoisatin (~40 µM, 100 µM and 200 µM) showed a significant induction of apoptosis (Figure 4f), but without any significant increase in the release of LDH compared to the DMSO control (Figure 4e). At the highest concentrations of 6-bromoisatin (~1000 µM and 2000 µM) Caco2 cells underwent a significant increase in LDH release (Figure 4e) with no in increase in caspase-3 and -7 activity.

Our results showed that 6-bromoisatin increased the level of caspase 3/7 in both cell lines, while tyrindoleninone only up-regulated the caspase 3/7 in HT29 cells. 6-Bromoisatin also showed more potency than tyrindoleninone producing higher levels of caspase 3/7 in HT29 cells and indicating high induction of apoptosis in these cells. The morphology of condensed chromatin and haloed areas in nearly all cells from the images was also consistent with this type of cell death. Furthermore, Caco2 cells treated with semi-purified 6-bromoisatin also underwent the induction of apoptosis. Therefore, semi-purified 6-bromoisatin in our study had the most consistent anti-cancer efficacy against both colon cancer cell lines at low concentrations. Necrosis, as indicated by LDH release, was only significantly increased with exposure to the highest concentrations of 6-bromoisatin in both cell lines. Our caspase 3/7 and LDH results suggest that 6-bromoisatin induces cell death by apoptosis at low concentrations, while the apoptotic pathway is terminated at higher concentrations and secondary necrosis or necrosis is being triggered [43,44]. It has been shown that some structurally similar isatin and indole compounds at low concentrations induce apoptosis through the activation of caspase 3 in a range of cell lines [28,45,46]. For example, caspase 3/7 was activated by 5,6,7-tribromoisatin at a concentration of 8 μM in the Jurkat cell line after 5 h [28]. Edwards *et al*. [27] showed that caspase 3/7 was up-regulated significantly with approximately 22 μM 6-bromoisatin in KGN cells and apoptosis was also confirmed by Tunnel staining in these cells.

Our results suggest that both tyrindoleninone and semi-purified 6-bromoisatin induce apoptosis through caspase-dependent pathways on HT29 cells. However, more investigation on initiator caspase 8 and 9 would be required to distinguish between the extrinsic and intrinsic apoptosis pathways [36,47] induced by these brominated indoles. In a review by Vine *et al.* [39], the mode of action of some halogenated isatins, such as 6-bromoisatin, was proposed to be linked to the reduction in extracellular signal-regulated protein kinase (ERK) activity. Another study by Cane *et al*. [48] suggests that isatin and indole inhibit cell proliferation and induce apoptosis via inhibiting the signaling of ERK. Inhibition of ERK can suppress cell growth and results in induction of apoptosis in the cells [49]. Moreover, some other apoptosis pathways, including both caspase-dependent or caspase-independent, can occur via inhibition of ERK, as has been reported by Georgakis *et al*. [50]. ERK may also act through suppression of the anti-apoptotic signaling molecule Akt [51]. Therefore, further study on ERK and Akt inhibition, especially with pure 6-bromoisatin, is required to evaluate the exact mode of action of these brominated compounds.

#### 2.2.2. Cell Cycle Analysis

**Figure 7:**
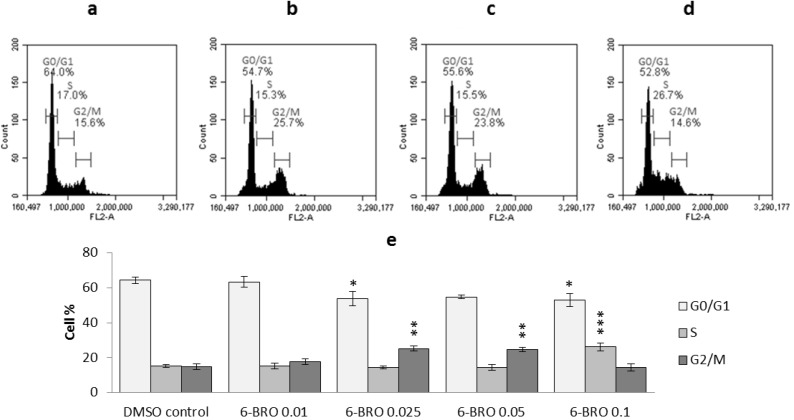
Cell cycle analysis using propidium iodide (PI) staining and flow cytometry. HT29 cells (5 × 10^5^ cells in 1 mL media/well) were treated for 12 h with (**a**) DMSO only (final concentration 1%); (**b**) 0.025 mg/mL 6-bromoisatin; (**c**) 0.05 mg/mL 6-bromoisatin; (**d**) 0.1 mg/mL 6-bromoisatin semi-purified from egg mass of *D. orbita*; (**e**) Results are the mean ± SE of three separate experiments. Significant difference between each group and the DMSO control are shown as *p* ≤ 0.05 (*); *p* ≤ 0.01 (**) and *p* ≤ 0.001 (***).

Cell cycle analysis revealed three distinct cell populations in HT29 cells, which were indicative of cells in the G0/G1, S and G2/M phases of the cell cycle (Figure 7). The DMSO control showed more accumulation of the cells in G0/G1 (64% ± 1.9%) with approximately the same proportion of the cells in S and G2/M (17% *versus* 15.6%). After exposure to ~400 µM (0.1 mg/mL semi-purified) 6-bromoisatin, 26.7% of HT29 cells were in the S phase (*p* ≤ 0.001). This switched to significantly more cells in G2/M at the lower and most effective concentrations (100 µM = 25.7% and 200 µM = 23.8%). There were no significant differences in the cell population analysis between the DMSO control negative and the cells treated with 6-bromoisatin at the concentration of 0.01 mg/mL. Our result revealed that the most effective concentration of 6-bromoisatin that induced the highest apoptosis in HT29 cells, also caused the accumulation of cells at G2/M phase of the cell cycle. G2 phase in the cell cycle is where DNA repair might occur in cells, along with preparation for mitosis in M phase [52].

Increasing arrest of the cells in G2/M phase has been shown to be associated with enhanced apoptosis [10]. CDK1 (cyclin dependent kinase) is one of the protein kinase families that is activated by dephosphorylation and acts as a G2 checkpoint, which controls cell cycle progression from G2 to M phase [52]. For example, in a study by Singh *et al*. [53] sulforaphane, a naturally occurring cancer chemopreventive agent, caused an irreversible arrest in the G2/M phase of human prostate cancer cells (PC-3), which was associated with a significant reduction in protein levels of cyclin B1, CDC25B, and CDC25C. In a study by Vine *et al*. [45] various *N*-alkyl isatins induced G2/M cell cycle arrest. It is known that the indole based small molecules inhibit serine/threonine kinases, glycogen synthase kinase-3 (GSK3) [30,54] and CDK5 [55,56]. Another well-known isatin derivative 6,6′-dibromoindirubin has also been identified as a specific GSK-3 inhibitor [29]. Anti-proliferative activity of indirubin has been shown via ATP-competitive inhibition of both CDK1 and CDK2 [57,58,59]. The modes of action associated with indirubins [58] includes the induction of apoptosis through cell cycle arrest at G2/M via the inhibition of GSK3 [30], as well as induction of the c-Src kinase and nuclear factor-κB signaling pathway and expression [60,61] and activation of the aryl hydrocarbon receptor [62,63]. Vine [37] tested the inhibitory effect of six representative *N*-alkyl isatins on a range of tyrosine-specific and serine/threonine-specific protein kinases, but found no inhibition of enzyme activity by these isatins [37]. Based on molecular modeling results, neither 6-bromoisatin or tyrindoleninone are predicted to have any kinase receptor binding or enzyme inhibiting activity [25]. However, inhibition of tubulin polymerisation in a range of cancer cell lines was shown by an array of imidazole and pyrrole containing 3-substituted isatins, resulting in cell cycle arrest at G2/M and final cell death [64,65]. Based on morphological examination of treated cells, Vine *et al*. [45] suggested that *N*-alkyl isatins may either stabilize or disrupt microtubules in a similar manner. Therefore, the finding that 6-bromoisatin increases the proportion of cells in the G2/M phase is consistent with a range of other studies on isatin derivatives and could be linked to a range of different modes of action that require further investigation.

## 3. Experimental Section

### 3.1. Egg Mass Extraction, Purification

All chemicals, HPLC grade solvents and silica gel where obtained from Sigma-Aldrich Pty Ltd. (Castle Hill, Australia) unless otherwise stated. *D. orbita* egg capsules (27 g) were collected from a recirculating aquarium in the School of Biological Sciences, Flinders University, South Australia. The eggs capsules were opened and soaked in 100 mL (per 10 g eggs) chloroform and methanol (1:1, v/v) under agitation at room temperature for 2 h, followed by overnight soaking in fresh solvent. Both extracts were combined and filtered. Then a low volume of milli-Q water (~20–30 mL) was added to facilitate the separation of methanol and chloroform into two phases. The chloroform layer was separated and dried under reduced pressure of 474 mbar on a Buchi rotary evaporator at 40 °C. The dried extracts were re-dissolved in a small volume of dichloromethane (~1 mL), transferred to amber vials, then dried under a stream of nitrogen gas, yielding 300 mg of a light brown/red oily extract which was subsequently stored at −20 °C. Previous research has shown that the dominant compounds in *D. orbita* extracts are colored and can be separated by silica chromatography [18]. Here flash column chromatography pressurized with nitrogen gas was used to separate the bioactive compounds. The stationary phase consisted of approximately 20 g silica gel (100 mesh) mixed with hexane. The chloroform extract (300 mg) was loaded onto the column and eluted using a stepwise gradient of solvents, starting with 100% hexane (100 mL, Fraction 1). Fraction 2 was eluted using 20% DCM in hexane (50 mL), then Fraction 3 was collected using 25% DCM in hexane (200 mL), followed by Fraction 4 with 100% DCM (200 mL). The polarity of the solvent was then increased to 10% methanol in DCM to collect Fractions 5 (15 mL) and 6 (85 mL). Finally, Fraction 7 was collected by washing the column with 50 mL 100% methanol. All solvents were evaporated from the fractions under reduced pressure by rotary evaporation at 40 °C.

### 3.2. Chemical Analysis

All fractions affecting cell viability in the MTT assay (see below) were further analyzed using liquid chromatography coupled with mass spectrometry (LC/MS). Briefly, fractions were dissolved in acetonitrile and analyzed by HPLC (Waters Alliance) that was coupled to a mass spectrometer (MS, Micromass, Quatro micro™) with a Hydro-RP C18 column (250mm × 4.6 mm × 4 μm) and parallel UV/Vis diode-array detection at 300 and 600 nm. The flow rate was 1 mL/min of formic acid and a gradient of acetonitrile in water, according to the methods established by Westley and Benkendorff [24]. Compounds were identified using electrospray ionization-mass spectrometry (ESI-MS) with a flow rate of 300 μL/min. Mass Lynx 4.0 software was used to analyze the data. Additional analysis on bioactive fractions was facilitated by gas chromatography–mass spectrometry (GC-MS, Agilent Technologies (Mulgrave, Australia) 5975C Series GC/MS) with a capillary column (SGE HT-5, 15 m × 0.25 mm i.d.) with a 0.25 µm film thickness. The injection port temperature was set at 260 °C. The initial oven temperature was held at 50 °C for 3 min and then ramped with a rate of 15 °C/min to the final temperature of 300 °C and held for 2 min. The carrier gas was helium with a constant flow rate of 2 mL/min. Electron ionisation (EI) was used with the electron energy of 70 eV. The source temperature was set to 230 °C and the MS quadrupole was 150 °C. To confirm the identity of the bioactive compounds, ^1^H NMR spectroscopy was also used on purified fractions on a Bruker Avance III 400 MHz spectrometer (Bruker Biosciences, Preston, Australia), operating at 294K, in deuterated acetonitrile. Chemical shifts (δ) are reported as parts per million (ppm) and referenced to residual solvent peaks. Spin multiplicities are indicated by: s, singlet; bs, broad singlet; d, doublet; t, triplet; q, quartet; m, multiplet; and dd, doublet of doublets.

### 3.3. Cell Culture

Two human colorectal cancer cell lines Caco2 (passage no. 26–34) and HT29 (passage no. 18–26) maintained at 37 °C in a 5% CO_2_ humidified atmosphere. The cells were cultured in Dulbecco’s Modified Eagle’s Medium (DMEM) supplemented with 4500 mg/L l-glutamine, 10% FBS, 100 U/mL Penicillin/Streptomycin and 1% Non-essential Amino Acid (100×).

### 3.4. MTT Viability Assay and Cell Morphology

All fractions and purified compounds were tested using an MTT viability assay which measured the reduction of MTT tetrazolium salt to formazan [66,67]. Caco2 and HT29 cells were grown to 70% confluence, detached from flasks with 1X Trypsin-EDTA, counted using trypan blue dye exclusion method, and plated into 96-well plates (Costar^®^) (2 × 10^4^ cells in 100 μL media/well). The cells were incubated for 48 h before treatment. All extracts and purified compounds were dissolved in 100% dimethylsulphoxide (DMSO) then diluted in media and added to the cell cultures in triplicate (final DMSO concentration of 1%), with final concentrations ranging from 2 to 0.01 mg/mL. 1% DMSO controls were also included on each plate. All extracts were incubated with the cells for 12 h. The media was removed prior to adding 100 µL of 0.05% MTT with fresh media to each well. The cells were incubated for 1 h and then 80 µL of 20% SDS in 0.02 M HCl was added to each well. The absorbance of the samples was determined spectrophotometrically after 1 h by measuring the optical density at 480 and 520 nm on a FLUOstar Omega microplate reader (BMG Labtech, Mornington, Australia). This assay was repeated on three separate occasions (*n* = 3). The morphological changes in HT29 cells were also observed by Olympus (Mt Waverly, Australia) CK2 inverted optical microscope (original magnification 400×) 12 h after treatment.

### 3.5. Combined Caspase 3/7, Membrane Integrity and Cell Viability Assays

HT29 and Caco-2 cells (2 × 10^4^ cells in 100 μL media/well) were seeded into sterile white (opaque) 96-well plates (Interpath, Heidelberg West, Australia) (for determination of apoptosis and necrosis) and clear sterile 96-well plates (Costar^®^) (for measurement of cell viability). All cells were incubated for 48 h to allow attachment of these adherent cells, then the media was removed and the cells were washed with PBS. The cells were treated with different concentrations of crude extract and purified compounds from 0.5 to 0.01 mg/mL in fresh media. Two positive controls were added to each plate in triplicate wells; staurosporin (5 µM/mL) for apoptosis and lysis solution (5 μL/well, Promega, Madison, WI, USA) for necrosis. All cells were treated for 12 h. To measure necrosis, 70 μL of supernatant from each well of the white opaque plate was transferred to another white opaque 96-well plate. The CytoTox-ONE Homogeneous Membrane Integrity Assay reagent (Promega) was applied based on the manufacturer’s instructions, in equal volume to the cell culture medium (70 μL). The plates were then incubated at 22 °C for 10 min and the fluorescence recorded with an excitation wavelength of 535 nm and an emission wavelength of 590 nm on a FLUOstar Omegaplate reader (BMG Labtech, Mornington, Australia). To measure apoptosis, the Caspase-Glo 3/7^®^ assay (Promega) was applied. 30 μL Caspase-Glo^®^ 3/7 Reagent was added to the primary white opaque 96-well containing cells and 30 μL cell culture medium and incubated at 22 °C for 1 h. The plates were read on a FLUOstar Omega with full light to capture total luminescence. This experiment was repeated on three separate occasions (*n* = 3).

### 3.6. Flow Cytometric Detection of Apoptosis

To confirm the caspase assay results, the most bioactive compounds were used in flow cytometry. HT29 cells were plated in 24 well plates (Nunc^®^) in duplicate with 1.5 × 10^5^ cells/well in 1 mL media, then incubated for 48 h. Media were removed and 1 mL media and treatments including 0.025 and 0.05 mg/mL semi-purified 6-bromoisatin and 0.05 mg/mL tyrindoleninone (final concentration of 1% DMSO) were added to each well. Staurosporin (5 µM/mL) was used as a positive control reagent for triggering apoptosis (data not shown). Cells were treated for 12 h and collected from the wells after the trypsinization by 1× trypsin-EDTA, then were placed in 15 mL tubes before centrifugation (1500 rpm for 3 min). Media were removed and the cells were washed twice with sterilized phosphate buffered saline (PBS) and suspended in 1× Binding buffer (10 mM Hepes/NaOH, pH 7.4, 140 mM NaCl, 2.5 mM CaCl_2_) at a concentration of 1 × 10^6^ cells/mL. 100 µL of the solution (1 × 10^5^ cells) were transferred to a 5 mL culture tube then 5 µL of FITC Annexin V (BD Biosciences, Franklin Lakes, NJ, USA) and 5 µL of propidium iodide (BD Biosciences) at 10 µg/mL final concentration were added to each tube. All cells were incubated for 15 min at RT (25 °C) in the dark and cell distribution was analyzed using FACSan Flow Cytometer (Becton Dickinson, North Ryde, Australia) and FlowJo analysis software.

### 3.7. Cell Cycle Analysis

Flow cytometry was used to assess whether the bioactive compounds arrested the cells at a particular stage of the cell cycle. HT29 cells (5 × 10^4^ cells in 1 mL media/well) were seeded into 12-well plates (Costar^®^). The cells were incubated for 48 h before treating with different concentrations of semi-purified 6-bromoisatin for 12 h (final DMSO concentration of 1%). The supernatant and cells were then harvested by exposing the cells to 0.25%, Trypsin-EDTA solution for 10 min, then centrifuged and washed in phosphate buffered saline (PBS), fixed in 3 mL ice-cold 100% ethanol and stored overnight at −20 °C. At the time of analysis, the cells were centrifuged, washed once again in PBS and stained with a freshly made solution containing 0.1 mg/mL propidium iodide (PI), 0.1% Triton x-100 and 0.2 mg/mL ribonuclease A in PBS. All samples were incubated for 30 min at room temperature in the dark. Cell cycle distribution was determined by an analytical DNA flow cytometer (Accuri C6, BD Biosciences) and CFlow Plus software on DNA instrument settings (linear FL2) on low.

### 3.8. Statistical Analysis

Statistical analyses were performed using SPSS and values of *p* ≤ 0.05 were considered to be statistically significant. One way ANOVA test was performed to compare between different concentrations of treatments and control. Tukey post-hoc test was applied to detect which groups significantly differ.

## 4. Conclusions

Our study demonstrated that both semi-purified 6-bromoisatin and purified tyrindoleninone decreased cell viability in the colon cancer cell lines HT29 and Caco2. In particular, 6-bromoisatin showed more specificity and potency than tyrindoleninone and greater induction of apoptosis toward the colon cancer cells. 6-Bromoisatin also inhibited cell cycle progression of HT29 cells by arresting some cells in the G2/M phase. This data, along with the previously reported *in-vivo* induction of apoptosis in DNA damaged cells of the colon using Muricidae extracts [22] suggests that 6-bromoisatin from Muricidae molluscs is promising as an anti-cancer drug against colon cancer.

## Supplementary Files

Supplementary File 1 3D structure (MOL, 1 KB)

Supplementary File 2 MOL-Document (MOL, 1 KB)

Supplementary File 3 3D structure (MOL, 2 KB)

Supplementary File 4 MOL-Document (MOL, 2 KB)

Supplementary File 5 3D structure (MOL, 2 KB)

Supplementary File 6 MOL-Document (MOL, 2 KB)

Supplementary File 7 3D structure (MOL, 1 KB)

Supplementary File 8 MOL-Document (MOL, 1 KB)

Supplementary File 9 3D structure (MOL, 3 KB)

Supplementary File 10 MOL-Document (MOL, 3 KB)
